# Evolution into Takayasu arteritis in a patient presenting with acute pulmonary oedema due to severe aortic regurgitation; a case report

**DOI:** 10.1186/s41927-018-0028-5

**Published:** 2018-07-12

**Authors:** Nipun Lakshitha de Silva, Milinda Withana, Praveen Weeratunga, Prakash Priyadharshana, Inoshi Atukorala

**Affiliations:** 1 Professorial Unit in Medicine, National Hospital of Sri Lanka, Colombo, Sri Lanka; 2 Institute of Cardiology, National Hospital of Sri Lanka, Colombo, Sri Lanka; 3 Department of Clinical Medicine, Faculty of Medicine, University of Colombo, Colombo, Sri Lanka; 4 Department of Clinical Medicine, Faculty of Medicine, General Sir John Kotelawala Defence University, Ratmalana, Sri Lanka

**Keywords:** Takayasu arteritis, Aortic regurgitation, Acute heart failure, Pulmonary oedema, Infective endocarditis

## Abstract

**Background:**

Takayasu arteritis is a rare large vessel vasculitis which predominantly affects young Asian females. Aortic regurgitation and heart failure are well described manifestations which are usually preceded by constitutional symptoms, limb claudication, pulse and blood pressure discrepancies, vascular bruits and features of organ ischaemia.

**Case presentation:**

A 25-year- old Sri Lankan female presented with a three days history of acute shortness of breath, cough and orthopnoea. On examination she had severe aortic regurgitation resulting in high output cardiac failure. There was no evidence of acute coronary ischaemia or infective endocarditis. The only significant investigation finding was an elevated erythrocyte sedimentation rate (ESR) of 114 mm/first hour. The patient was treated for pulmonary oedema and empirically for infective endocarditis. Extensive evaluation for an underlying infection, large vessel vasculitis or malignancy did not reveal any abnormalities. Detailed periodic assessment identified reduced blood pressure in left arm (70/40 mmHg) compared to right (100/70 mmHg) and reduced pulse volume of left arm with left subclavian bruit more than one year after the initial presentation. Digital subtraction angiography revealed significant stenosis at first part of left subclavian and origin of left vertebral arteries. A diagnosis of Takayasu arteritis was made and patient was started on high dose glucocorticoids.

**Conclusions:**

Takayasu arteritis can present initially with isolated cardiac involvement even as acute cardiac manifestations and high degree of suspicion with close follow up would allow early detection of development of other classic features and timely diagnosis.

## Background

Takayasu arteritis is an uncommon large vessel vasculitis which predominantly affects the aorta and its main branches [1]. It causes chronic granulomatous inflammation and necrosis of the vessel wall culminating in vessel wall thickening, fibrosis, stenosis and thrombus formation. This disease occurs commonly in young females and is predominantly seen among Asians though disease is reported worldwide [2].

Clinical manifestations of Takayasu arteritis are due to organ ischemia. These findings vary from asymptomatic incidental diagnoses to catastrophic neurological and cardiovascular manifestations. Common clinical features include constitutional symptoms such as fever, malaise weight loss, symptoms due to arterial occlusion, hypertension and cardiac involvement [3]. Acute pulmonary oedema due to aortic regurgitation is a recognized manifestation of Takaysu arteritis, but this being the only clinical presenting feature is not reported. We report a young female who presented with acute pulmonary oedema due to aortic regurgitation without any other clinical features of Takayasu arteritis, subsequently diagnosed as Takayasu arteritis after a long follow up period.

## Case presentation

A 25- year-old Sri- Lankan female presented with progressively worsening shortness of breath, orthopnoea and dry cough for three days. Her symptoms were not associated with chest pain, fever, oedema or wheezing. At the onset she noted shortness of breath on mild exertion which progressed to shortness of breath even at rest by third day. The patient reported a good urine output throughout. She was asymptomatic prior to the onset of this illness excepting a mild iron deficiency anaemia for which she was on oral iron treatment and dysmenorrhoea attributed to an ovarian cyst. The patient had undergone an appendicectomy eight months prior to the presentation for appendicitis associated with round worm infestation.

Examination revealed dyspnoea at rest, elevated jugular venous pulse, tachycardia with a heart rate of 120/min, blood pressure of 100/70 mmHg on both arms. There was an early diastolic murmur over left sternal edge associated with a thrill and bilateral lower lung field crackles. Rest of the examination was normal. Her estimated body surface area was 1.24m^2^ with a body weight of 40 kg and height of 140 cm.

Investigations revealed white cell count of 7.1 × 10^9^/l (4–11 × 10^9^/l), platelet count of 435 × 10^9^/l (150–400 × 10^9^/l),haemoglobin of 10.9 g/dl (11.5–15.5 g/dl) with mean corpuscular volume of 78 fl (80-96 fl). C- reactive protein was 18 mg/l (< 6 mg/l) and Erythrocyte sedimentation rate (ESR) was 114 mm/1^st^ hour (< 20 mm/1^st^ hour). Renal and liver functions, urinalysis, blood sugar and coagulation profile were in normal range. Electrocardiogram revealed sinus tachycardia with no ischaemic changes. Troponin I was negative. Chest radiograph had findings consistent with pulmonary oedema. Repeated blood cultures were negative. Transthoracic echocardiogram demonstrated ejection fraction > 60%, severe aortic regurgitation with dilated aortic root when adjusted for her body surface area(annulus 21 mm, sinus 34 mm). Aortic valve leaflets were morphologically normal. There was no left ventricular hypertrophy, regional wall motion abnormalities or vegetations. Other cardiac valves were normal. All four cardiac chambers were of normal size. Transoesophageal echocardiogram confirmed the absence of vegetations.

She was started on supportive management for acute pulmonary oedema and treatment was initiated as for infective endocarditis empirically. Though she was referred to cardiac surgery team urgent surgical interventions were not performed since she improved with medical management. After completion of four weeks empiric antibiotics her echocardiographic changes and ESR remained unchanged. However, heart failure improved with medical management with complete resolution of orthopnoea and dyspnea being limited to moderate exertion. Intravenous antibiotics were omitted in the absence of convincing evidence of endocarditis.

Further evaluation was performed in view of persistently high ESR and aortic regurgitation with no definitive cause. Chronic infections such as tuberculosis, vasculitic conditions such as Takayasu arteritis were considered. Further questioning did not reveal constitutional symptoms, contact with tuberculosis, arm claudication, headache or any neurological symptoms. Examination did not reveal any lymphadenopathy, hepatosplenomegaly, cutaneous or joint involvement, fundoscopic abnormalities, pulse deficit or vascular bruits. Rheumatoid factor, serum cryoglobulins, anti nuclear antibodies and complement levels were normal. Peripheral blood smear revealed rouleaux formation with evidence of mild iron deficiency anaemia. Serum protein electrophoresis and serum Lactate dehydrogenase were normal. Human Immunodeficiency Virus antibodies and Serology for syphilis were negative. Mantoux test and induced sputum for acid fast bacilli were negative. Ultrasound abdomen, Contrast enhanced Computed tomography (CT) of the chest, abdomen and pelvis as well as CT aortogram with arch vessels were normal except mild aortic root dilatation. Bone marrow biopsy revealed reactive marrow with no other abnormalities while bone marrow culture for bacteria, fungi, mycobacteria, brucella and leishmania were negative. Colonoscopy and biopsy did not reveal any abnormality.

**Fig. 1:**
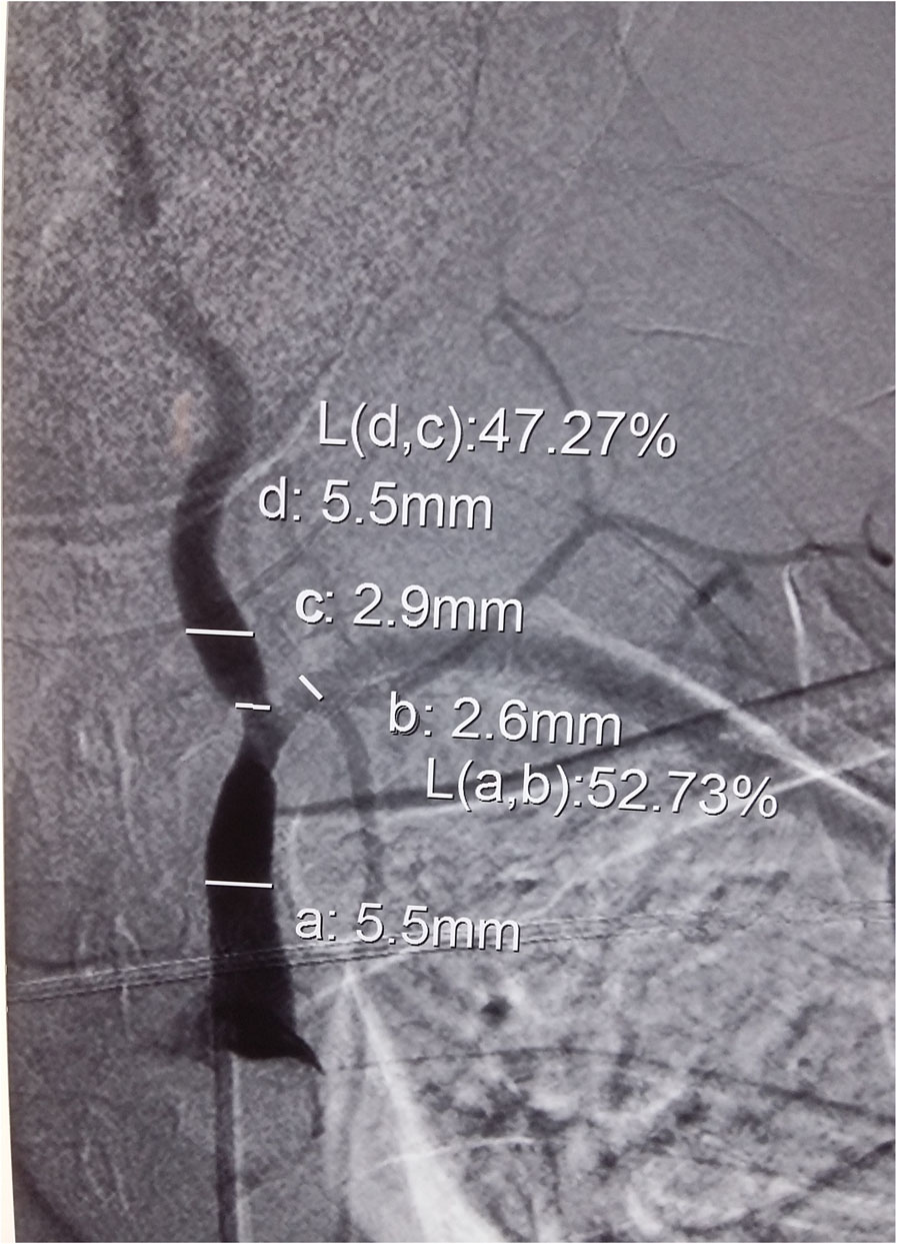
Digital subtraction angiography of left subclavian artery of the patient. Digital subtraction angiography of the patient performed sixteen months after the initial presentation revealed significant stenosis of the left subclavian artery and stenosis at the origin of left vertebral artery. Other major branches of aorta including renal arteries were normal

The patient was followed up for one year with detailed clinical assessment and continuation of medical management of heart failure. She did not develop any new symptoms and her exertional dyspnoea remained static. Her ESR remained above 100 mm/1^st^ hour. Clinical examination about one year after initial presentation revealed a reduced pulse volume of left upper limb with a blood pressure difference (right- 100/70 mmHg, left- 70/40 mmHg). There was a left subclavian bruit as well. But, patient did not have any constitutional symptoms or any symptoms of left upper limb ischaemia. Digital subtraction angiography at that point revealed significant stenosis at first part of left subclavian artery and at the origin of left vertebral artery (Fig. 1). Based on the new findings she was diagnosed to have Takayasu arteritis and was started on prednisolone 1 mg/kg body weight daily with plan for follow up at cardiology and rheumatology units. Six weeks after initiation of glucocorticoids patient remained clinically well and ESR decreased to 25 mm/1^st^ hour. Glucocorticoid dose was slowly tapered. Decision on aortic valve replacement was decided to be made few months later after resolution of active inflammation and repeat cardiac assessment. Ravascularisation for arterial stenosis was not offered in the absence of symptoms of upper limb or cerebral ischaemia. Sequence of events from her presentation to the diagnosis is given in a timeline in Table 1.

**Table 1:** Timeline of events with diagnostic tests and interventions

Date of illness	Events	Diagnostic tests	Interventions
18/10/2016	Onset of shortness of breath, cough		
21/10/2016	Admission		Supportive management of acute heart failure
22/10/2016		ESR- 114 mm/1st hour CRP- 18 mg/l Echocardiogram- severe aortic regurgitation	Emperical intravenous antibiotics
11/11/2016	Persistent aortic regurgitation, symptomatic improvement in heart failure	Transoesophageal echo- no vegetation	
20/11/2016	Completion of antiobitics	ESR- 102 mm/1^st^ hour Echocardiogram- severe aortic regurgitation	Evaluation for other aetiology
25/11/2016		CT aortogram and angiogram- normal	Continued medical management of heart failure
27/10/2017	Detection of left subclavian bruit, blood pressure and pulse difference	Digital subtraction angiogram- significant stenosis of first part of left subclavian and origin of left vertebral arteries	Initiation of glucocorticoids

## Discussion and conclusions

The initial clinical presentation of our patient with acute pulmonary oedema with aortic regurgitation with elevated inflammatory markers led to the clinical suspicion of infective endocarditis. But patient did not have any convincing evidence of infective endocarditis and was treated empirically which did not provide significant improvement. As a result we evaluated for an alternative diagnosis for the initial constellation of clinical features. Due to the combination of high inflammatory markers in a young Asian female with unexplained aortic regurgitation with mild aortic root dilatation a diagnosis of Takayasu arteritis was considered. But thorough workup did not reveal any other clinical features and initial CT- angiogram did not show any arterial involvement. Magnetic resonance angiography (MRA) is considered to be superior to CT angiogram in detecting early vascular changes such as increased mural vascularity and mural oedema [4]. In our patient if we had access to MRA there is a possibility of establishing the diagnosis early. Positron emission tomography (PET) scan would have revealed arterial wall inflammation early if it was utilized in evaluation of this patient [5]. However due to lack of availability of this investigation we could not perform on our patient.

With the emergence of new examination findings again the diagnosis of Takayasu arteritis became more likely and was further confirmed by angiography results. In the absence of single diagnostic test for Takayasu arteritis, diagnosis is made based on suggestive clinical picture and imaging findings. American College of Rheumatology criteria are used mainly for classification in research purposes, however our patient fulfills five out of six criteria for the diagnosis of Takayasu arteritis [6].

Cardiovascular manifestations of Takayasu arteritis include aortic regurgitation, pulmonary hypertension, ischaemic heart disease due to coronary artery involvement and left ventricular hypertrophy due to hypertension [1, 7]. Direct myocardial involvement and pericardial effusions have also been reported [3]. Aortic regurgitation is thought to be due to dilatation of the ascending aorta, separation of the aortic valve leaflets and valve thickening [1]. Heart failure can occur due to aortic regurgitation, ischemic heart disease, myocarditis and chronic hypertensive heart disease. Most of the available large case series describe these cardiac manifestations in a significant proportion of patients with valvular heart disease occurring in more than 20% patients out of which aortic valve involvement contributing to the majority [7, 8]. Cardiac failure is also known to occur in about 10–30% of the patients depending on the case series, but the symptoms are commonly described together with other vascular and systemic features.

Classically described Takayasu arteritis has several phases in which initial inflammatory stage is followed by latter stenotic stage. Early or pre-pulseless phase is characterized by non specific systemic features including fever, malaise, athralgia, myalgia followed by pulseless or vascular inflammatory phase where there is limb claudication, renovascular hypertension and gastrointestinal and skin changes. Stenotic phase is the last stage where there is vessel stenosis, aortic regurgitation and heart failure [9]. According to this classic description, heart failure and aortic regurgitation are expected to occur late in the course of illness unlike our patient. Acute heart failure in isolation due to aortic regurgitation is further uncommon.

There are several case reports where patients have presented with heart failure in Takayasu arteritis. One recent case report describes a teenage girl who presented with acute heart failure due to myocarditis, however she had preceding arm claudication, systemic and cutaneous manifestations in the preceding one year [10]. In another case report, a 27-year-old female who presented with features of heart failure was found to have severe pulmonary hypertension and dilated cardiomyopathy together with pulse and blood pressure difference between two arms [11]. This patient also did not have any constitutional symptoms. Another case report describes a 9-year-old girl presenting with acute heart failure due to severe aortic stenosis with further evaluation revealing absent lower limb pulses and abnormal angiography [12]. Even the available case reports of heart failure due to aortic regurgitation describe concomittant presence of other features of vascular occlusion [13, 14].

Our patient presented with initial isolated aortic regurgitation and high output cardiac failure and subsequent development of subclavian artery involvement on examination and imaging yet without associated symptoms provide a unique case history. The clue to the detailed periodic examination for an underlying aetiology was the persistently high ESR. Patients with AR in Takayasu arteritis have a poor outcome [15] and surgical repair poses difficulties due to associated inflammation. Therefore, early diagnosis is crucial.

This indicates that Takayasu arteritis can present differently from classically described phases of illness and without any systemic constitutional symptoms or typical features of vascular occlusion. Therefore, careful evaluation and periodic assessment would minimize the delay in diagnosis and initiation of therapy.

## Abbreviations

CT: Computed tomography
ESR: Erythrocyte Sedimentation Rate
MRA: Magnetic resonance angiography

## References

[1] Johnston S, Lock R, Gompels M (2002). Takayasu arteritis: a review. J Clin Pathol.

[2] Onen F, Akkoc N (2017). Epidemiology of Takayasu arteritis. La Presse medicale.

[3] Li J, Sun F, Chen Z, Yang Y, Zhao J, Li M, Tian X, Zeng X (2017). The clinical characteristics of Chinese Takayasu's arteritis patients: a retrospective study of 411 patients over 24 years. Arthritis Res Ther.

[4] Nastri MV, Baptista LP, Baroni RH, Blasbalg R, de Ávila LF, Leite CC, de Castro CC, Cerri GG (2004). Gadolinium-enhanced three-dimensional MR angiography of Takayasu arteritis. Radiographics.

[5] Tezuka D, Haraguchi G, Ishihara T, Ohigashi H, Inagaki H, Suzuki J-i, Hirao K, Isobe M (2012). Role of FDG PET-CT in Takayasu arteritis: sensitive detection of recurrences. J Am Coll Cardiol Img.

[6] Arend WP, Michel BA, Bloch DA, Hunder GG, Calabrese LH, Edworthy SM, Fauci AS, Leavitt RY, Lie J, Lightfoot RW (1990). The American College of Rheumatology 1990 criteria for the classification of Takayasu arteritis. Arthritis Rheum.

[7] Soto M, Espinola N, Flores-Suarez L, Reyes P (2008). Takayasu arteritis: clinical features in 110 Mexican mestizo patients and cardiovascular impact on survival and prognosis. Clin Exp Rheumatol.

[8] Mwipatayi BP, Jeffery PC, Beningfield SJ, Matley PJ, Naidoo NG, Kalla AA, Kahn D (2005). Takayasu arteritis: clinical features and management: report of 272 cases. ANZ J Surg.

[9] Barto° D, Bonta° E, Ghiorghe S: Takayasu’s arteritis Arteritis–An update. Cercetãri Experimentale & Medico-Chirurgicale 2006;3:149–52.

[10] An X, Han Y, Zhang B, Qiao L, Zhao Y, Guo X, Fang L, Zhu W, Fang Q, Shen Z, Zhang S (2017). Takayasu arteritis presented with acute heart failure: case report and review of literature. ESC Heart Fail.

[11] Khan MA, Chandrakar SD, Shetty V (2016). Heart failure as the initial manifestation of takayasu’s arteritis. Int J Res Med Sci.

[12] Yang M-C, Yang C-C, Chen C-A, Wang J-K (2013). Takayasu arteritis presenting with acute heart failure. J Am Coll Cardiol.

[13] Kashima K, Kawasaki D, Yotsumoto G, Hatake S, Yamashita E, Nagayoshi S, Yoshishige Y, Tanoue K, Nagano S, Tanaka H (2010). Rapid progression of aortic regurgitation with thoracic aortic aneurysm due to Takayasu arteritis associated with ulcerative colitis. Intern med (Tokyo, Japan).

[14] Bolin E, Moodie DS, Fraser CD, Guirola R, Warren R, Eldin KW (2011). Takayasu arteritis presenting as severe ascending aortic arch dilation and aortic regurgitation in a 10-year-old female. Congenit Heart Dis.

[15] Morii S (1995). Follow-up study of Takayasu arteritis with aortic regurgitation. J Cardiol.

